# Acute Kidney Injury Post-Liver Transplant Using Grafts Treated with Hypothermic Machine Perfusion: From Biology to Surgical Aspects

**DOI:** 10.3390/ijms27031235

**Published:** 2026-01-26

**Authors:** Irene Scalera, Grazia Labellarte, Oronzo Ligurgo, Francesco D’Amico, Gianluigi Gigante, Stefania Roselli, Maria Filippa Valentini, Rossana Franzin, Alessandra Stasi, Loreto Gesualdo, Francesco Tandoi

**Affiliations:** 1 Hepatobiliary and Liver Transplant Unit, University Hospital Policlinic of Bari, 70124 Bari, Italy; grazia.labellarte@gmail.com (G.L.); oronzoligurgo@gmail.com (O.L.); francesco.damico1@uniba.it (F.D.); gainluigi.gigante@gmail.com (G.G.); stefaniaroselli@yahoo.it (S.R.); mariapiavalentini@gmail.com (M.F.V.); francesco.tandoi@uniba.it (F.T.); 2 Department of Precision and Regenerative Medicine and Ionian Area, University of Bari, 70121 Bari, Italy; loreto.gesualdo@uniba.it; 3 Nephrology, Dialysis and Transplant Department, University Hospital Policlinic of Bari, 70124 Bari, Italy; rossanafranzin@hotmail.it (R.F.); stasi.alessandra85@gmail.com (A.S.)

**Keywords:** acute kidney injury, machine perfusion, liver transplantation, ischemia–reperfusion injury

## Abstract

Many advantages have been reported with the use of machine perfusion (MP) to rescue extended criteria donor (ECD) grafts, improving both short- and long-term post-liver transplantation (LT) outcomes. Acute kidney injury (AKI) is a common post-LT complication associated with these grafts and may compromise patient outcomes and increase LT-related costs. The aim of the study was to analyze the incidence of AKI in recipients of MP-treated grafts compared with those receiving conventionally cold-stored (SCS) grafts, both before and after a propensity score matching (PSM). From a prospectively maintained database, LT recipients of MP-treated grafts were compared with SCS grafts transplanted in the same study period (January 2022–May 2025). PSM was performed based on donor risk index (DRI), macrosteatosis (≥ or <30%), and recipient NaMELD score using a 3:1 (MP vs. SCS) ratio. Of the 177 consecutive LTs, 30 were performed with MP-treated grafts (MP group) and 147 using SCS (SCS group). The MP group displayed more marginal characteristics: older age (72 vs. 62 years, *p* = 0.02), higher proportion of DCD (10% vs. 0, *p* = 0.04), and higher frequency of moderate steatosis (macro ≥ 30%, 10% vs. 2.7%, *p* = 0.09). AKI rates were similar between groups (63% vs. 45.6%, *p* = 0.16), as was the distribution across AKI stages. After PSM, donor and recipient characteristics were balanced, and AKI rates remained similar between groups (58.6% vs. 47.1%, *p* = 0.39). Donor diabetes and recipient age were independent predictors of AKI in multivariate analysis (donor diabetes OR 3.29, 95% CI 1.347–8.030; recipient age: OR 1.06, 95% CI 1.015–1.097, both *p* < 0.05). MCP-1 and TNF-α levels measured in the perfusate fluid within the first minutes of perfusion were positively correlated with post-LT creatinine peak (MCP-1, *p* = 0.00023, R = 0.58; TNF-α, *p* = 0.0004, R = 0.57). In conclusion, machine perfusion remains a valuable strategy for rescuing ECD liver grafts. In the current era—characterized by increasing use of machine-perfused grafts and extended criteria donors—recipients demonstrate postoperative renal outcomes comparable to those receiving conventionally preserved grafts.

## 1. Introduction

Acute kidney injury (AKI) is a critical complication after liver transplantation (LT) [1]. It can be associated with increased mortality [2,3], longer hospital stays, higher healthcare costs, and greater utilization of medical resources [4,5,6,7]. Moreover, AKI can progress to chronic kidney disease, complicating the management of the immunosuppressive therapy and increasing cardiovascular events [8,9].

The reported incidence varies across studies, and a meta-analysis estimated an overall incidence of 40.7%, with 7.7% representing severe AKI requiring renal replacement therapy (RRT) [10].

AKI arises from multiple factors, including donor characteristics, donation after circulatory death (DCD), graft ischemic times, recipient hepatorenal function and comorbidities, surgical factors, and postoperative therapies such as immunosuppression [6,11,12,13,14,15].

From a pathophysiological perspective, ischemia–reperfusion injury (IRI) is considered the main driver of AKI [16], and many donor-related risk factors have been linked to the severity of IRI. Severe steatosis, prolonged static-cold ischemia time, and DCD are strong predictors of poor post-LT liver function, and donors exhibiting these features were historically classified as “marginal” [17,18,19]. The underlying mechanisms are very complex and involve multiple interacting cell populations mediated by cytokines [19,20].

Machine perfusion, either normothermic or hypothermic, has demonstrated the ability to mitigate hepatic IRI damage, regenerating the graft before implantation and reducing post-LT liver-related complications. Therefore, MP has proven to be a valuable tool for expanding the donor pool and addressing the shortage of organs [21,22]. MP has pushed many donor boundaries, enabling the successful use of DCD grafts with long donor warm ischemia times [23] or those from very old donors [24,25]. However, moderate or severe macrovesicular steatosis still limits the broader use of these grafts [26].

Currently, the use of extended criteria donors (ECDs), regardless of the definition applied, is increasingly common. AKI remains a detrimental complication after implantation of these grafts [12], but it is not yet well established whether MP modifies the risk of post-LT AKI. While several studies have assessed MP in terms of liver function, morbidity, and mortality—including randomized trials—few have focused specifically on renal outcomes [27,28,29,30]. Noren et al. specifically evaluated the impact of hypothermic machine perfusion (HMP) on post-transplant renal outcomes; however, their study was limited to grafts from donation after brain death (DBD) donors aged ≥ 70 years and did not include additional extended donor criteria, such as steatotic grafts, donation after circulatory death (DCD), or donors with elevated body mass index (BMI). In that cohort, donor age did not adversely affect recipient renal outcomes, either in terms of AKI incidence or severity [31]. Similarly, a large cohort study focusing on elderly DBD donors reported a comparable incidence of stage 2–3 AKI and no difference in the need for renal replacement therapy (RRT) [25]. Moreover, two randomized trials involving extended criteria DBD grafts found no significant differences in RRT requirements; however, detailed data regarding AKI incidence and staging were not reported [29,32]. A large ongoing cohort study aims to predict post-LT graft failure at 90 and 365 days, including ECD grafts in the era of MP, but AKI still requires dedicated investigation [33].

Therefore, the aim of this study is to investigate the incidence and clinical impact of AKI in recipients of MP-treated grafts compared with recipients of SCS grafts. Given that machine perfusion is more frequently applied to extended criteria donor grafts, propensity score matching was performed to better balance donor and recipient characteristics. In addition, perfusate cytokine analyses were conducted to complement the clinical findings with biological data.

## 2. Results

From January 2022 to May 2025, 177 adult LT recipients were included; 30 (17%) received grafts treated with MP and 147 (83%) with SCS. Donor and recipient characteristics and surgical variables are summarized in Table 1.

**Figure 1:**
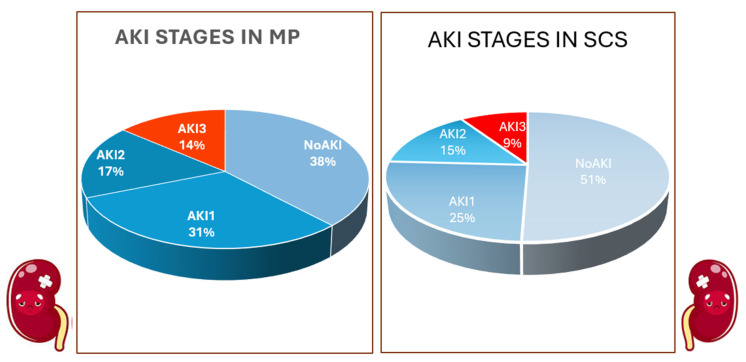
AKI stage distribution in the MP vs. SCS group after PSM analysis. (Abbreviations: AKI = acute kidney injury; MP = machine perfusion; SCS = static cold storage).

Before PSM, the MP group exhibited slightly more marginal donor characteristics compared with SCS: donor age was higher (72 vs. 62 years, *p* = 0.002), and 10% of MP grafts had macrovesicular steatosis > 30% compared with 2.7% in the SCS group (*p* = 0.09). As per institutional protocol, all DCD grafts underwent MP; thus, no DCD grafts were included in the SCS group. Recipients were similarly distributed between the groups, including age (58 vs. 57 years, *p* = 0.77), BMI (27 vs. 26 kg/m^2^, *p* = 0.69), MELD/NaMELD (16/18 vs. 15/16, *p* = 0.17), and pre-LT renal function (creatinine 1.28 vs. 0.97 mg/dL, *p* = 0.17). The etiology of liver disease was also comparable. Approximately one-third of patients had HCC before LT (37% vs. 33%, *p* = 0.86). A higher proportion of MP patients was transplanted for metabolic dysfunction-associated steatohepatitis (MASH) (40% vs. 26.5%, *p* = 0.24). Both groups predominantly underwent the piggyback technique (86.7% vs. 86.4%, *p* = 1.00). Postoperative outcomes are shown in Table 2. AKI incidence was higher in MP patients (63.3% vs. 45.6%), though not significantly (*p* = 0.16). The postoperative creatinine peak was significantly higher in MP compared to SCS patients (1.96 vs. 1.53 mg/dL, *p* = 0.02). AKI severity distribution (Figure 1) was similar between groups, with stage 1 being the most common (33.3% vs. 22.4%, *p* = 0.16), and only a small proportion had stage 3 disease (13.3 vs. 9.5, *p* = 0.29).

Other postoperative outcomes were comparable, including liver function (EAD 23.3% vs. 37.4%, *p* = 0.15), intensive care unit (ICU) stay (5 vs. 3 days, *p* = 0.26), total hospitalization (26 vs. 18 days, *p* = 0.17), and mortality (16.7% vs. 12.9%, *p* = 0.56).

After PSM, donor characteristics became balanced (Table 1), except for age (71 vs. 64 years, *p* = 0.02) and donor diabetes, which remains more frequent in the MP group (27.6% vs. 10.3%, *p* = 0.03). Moderate macrosteatosis was also more common in the MP group (10.3 vs. 4.6%, *p* = 0.02). Recipient characteristics (Table 1) and surgical variables were all similar in both groups. AKI rate after PSM remained similar (58.6% vs. 47%, *p* = 0.39) with comparable distribution of stages (stage 1: 31% vs. 25.3%; stage 2: 17.2% vs. 14.9%; stage 3: 13.2% vs. 9.2%, *p* = 0.69). EAD was slightly less frequent in MP recipients, although not significantly (24.1% vs. 41.4%, *p* = 0.07).

Univariate and multivariate analysis of the entire cohort identified donor diabetes and recipient age as independent predictors of post-LT AKI. The odds ratio for donor diabetes was 3.29 (95% CI = 1.347–8.030), and for recipient age, it was 1.06 (95% CI = 1.015–1.097) (Table 3).

**Figure 2:**
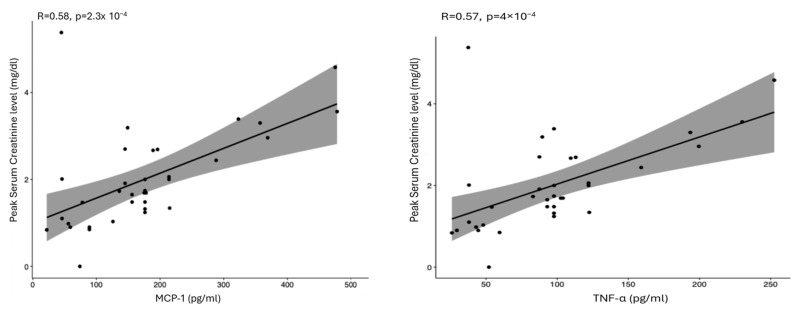
Correlation between postoperative creatinine peak and MCP-1 or TNF-α. (Abbreviations: MCP-1 = monocyte chemoattractant protein-1; TNF-α = tumor necrosis factor α).

**Figure 3:**
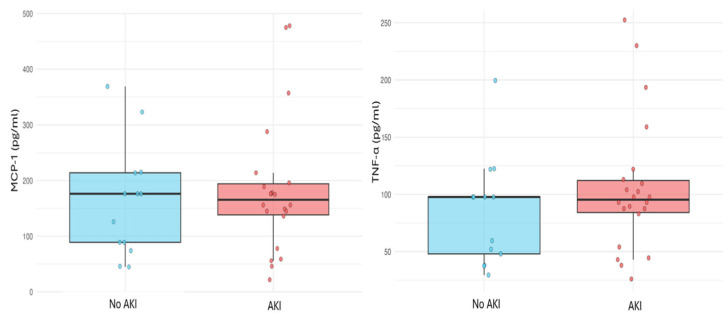
Correlation between AKI and MCP-1 or TNF-α. (Abbreviations: AKI = acute kidney injury; MCP-1 = monocyte chemoattractant protein-1; TNF-α = tumor necrosis factor α).

Spearman correlation showed a positive correlation between MCP-1/TNF-α levels and postoperative creatinine peak (MCP-1/creatinine: rho = 0.58, *p* = 0.0023; TNF-α/creatinine: rho = 0.57, *p* = 0.004) (Figure 2). No correlation was found between AKI incidence and cytokine levels (*p* < 0.05) (Figure 3).

## 3. Discussion

Machine perfusion is expected to mitigate ischemia–reperfusion injury. However, Noren et al. reported that 77% of recipients of MP grafts from elderly donors developed AKI, compared with 56% of recipients of SCS grafts—a clinically relevant difference, despite limited statistical significance. In their analysis, the relative risk for AKI was 1.36 (95% CI 1.02–1.80, *p* = 0.03) in MP grafts and 1.18 (95% CI 0.32–4.42, *p* = 0.80) for RRT [31]. Consistently, ECD grafts are known to be associated with a higher AKI risk [12]. Czigany et al. extensively investigated the postoperative outcomes after MP-preserved DBD grafts but without a specific focus on AKI [32]. In a large published cohort of elderly DBD donors preserved with MP, the rates of AKI stage 2–3 did not differ significantly compared with SCS (24% vs. 28%, *p* = 0.8) [25].

This study specifically investigated AKI after LT of MP-treated grafts. Despite the MP group receiving more ECD grafts (older, diabetic donors, greater steatosis, and DCD), early postoperative liver and kidney outcomes were comparable to those in the SCS group. After PSM, AKI incidence remained similar (Table 2). The distribution of AKI severity was also comparable—stage 1, 33.3% vs. 22.4%; stage 2, 16.7% vs. 13.6%; and stage 3, 13.3% vs. 9.5%—in the MP and SCS groups, respectively (*p* < 0.05) (Table 2). After PSM analysis, balancing donor and recipient characteristics of the set as much as possible, AKI was still not worse in the MP group compared with SCS (58.6% vs. 47.1%, *p* > 0.05) (Table 2). This result is noteworthy, as in the MP group, donors and grafts were still more marginal than SCS, even after PSM (Table 1, Figure 1). Importantly, this cohort included donors meeting multiple extended criteria, rather than only DBD and/or elderly donors. In the HMP group, 10 grafts were from DCD donors, approximately 30% of donors had diabetes, and donors were moderately overweight (mean BMI 28 kg/m^2^). Furthermore, the delayed introduction of tacrolimus until postoperative day 3 allowed us to reasonably exclude calcineurin inhibitor–related nephrotoxicity as a major contributor to AKI, thereby enabling a clearer assessment of donor- and recipient-related pre-transplant risk factors.

Notably, MP recipients consistently exhibited a higher creatinine peak. Although postoperative serum creatinine levels were significantly higher in the MP group, the incidence and severity of AKI did not differ between groups. This apparent discrepancy likely reflects a shift toward higher creatinine values that did not translate into clinically overt AKI according to established criteria. Given that machine perfusion was preferentially applied to marginal grafts, this finding may indicate a transient, subclinical renal dysfunction driven by early inflammatory and ischemia–reperfusion-related mechanisms, rather than an increased risk of clinically relevant kidney injury. Importantly, the comparable AKI rates suggest that machine perfusion may mitigate progression toward more severe renal impairment despite higher early creatinine levels. Given the KDIGO definition of AKI [34], this may be clinically relevant but not scientifically meaningful, and a transient rise in creatinine might influence early postoperative management (fluid balance, diuretics, and calcineurin inhibitor dosing).

This study highlighted new risk factors for AKI in the MP era. Donor diabetes and recipient age emerged as independent predictors of AKI (donor diabetes OR 3.29, *p* = 0.009; recipient age OR 1.06, *p* = 0.006) (Table 3). This emphasizes the relevance of vascular and metabolic donor factors in determining renal stress after LT, even when MP is used. Notably, surgical technical variables, including caval reconstruction and warm ischemia, were not associated with AKI. All patients transplanted with the classic technique underwent a total clamp of the vena cava without venovenous bypass before the implant, yet this did not influence AKI.

The correlation between perfusate cytokine expression (MCP-1 and TNF-α) and postoperative creatinine suggests a biologically plausible early biomarker for renal stress. Although cytokines were not associated with categorical AKI diagnosis, the biochemical correlation is compelling and warrants further investigation. (Figure 2 and Figure 3). Early identification of at-risk patients—before graft implantation—may allow optimized fluid management and immunosuppression tailoring. Recent studies have examined cytokine trends during normothermic MP cycles and postoperative outcomes, though not specifically AKI [35]. This is the first study proposing a potential pre-LT predictor of AKI sampled during MP. Further analyses are underway.

The main limitation of this study is the small sample of MP, as the analysis included the first consecutive grafts treated with machine perfusion. This represented an additional rationale for performing propensity score matching. Furthermore, despite the application of propensity score matching to enhance group comparability, residual imbalances in selected donor-related variables—namely donor age, donor diabetes, and the extent of moderate macrosteatosis—remained. This finding likely reflects the intrinsic clinical indication for machine perfusion, which is preferentially applied to extended criteria donor grafts. Consequently, even with rigorous matching strategies, complete elimination of baseline imbalances may not be achievable. Their potential impact on the observed outcomes cannot be entirely excluded and should be considered when interpreting the results. Moreover, only MCP-1 and TNF-α were analyzed, and their assessment required several working days. The evaluation of a broader cytokine panel, ideally with rapid turnaround times, may provide additional insights and enhance the clinical applicability of perfusate biomarker analysis in the short term.

Finally, this was a single-center study based on data from the early phase of the center’s experience. While this may have introduced some degree of selection bias in recipient allocation, it also represents a potential strength, as all procedures were performed according to a strictly protocol-driven approach and managed by a consistent anesthetic and surgical team.

## 4. Materials and Methods

### 4.1. Study Design

The study was conducted using data from a prospectively maintained database and retrospectively analyzed all recipients transplanted with grafts treated with MP. Initially, this group was compared with all adult patients who received SCS grafts during the same study period, from January 2022 to May 2025. AKI was assessed, and a multivariate analysis of risk factors for developing AKI was performed. Donor, recipient, and graft characteristics, as well as technical aspects, were compared between the two groups.

Cytokines, in particular monocyte chemoattractant protein-1 (MCP-1) and tumor necrosis factor α (TNF-α), were measured at the beginning of the MP treatment (T0) and correlated with AKI. MCP-1 and TNF-α were chosen because they play a significant role in acute kidney injury (AKI) following LT, and evidence suggests that they are key mediators of post-LT renal dysfunction [36,37]. They were measured by ELISA assay according to the manufacturer’s instructions. (TNF-α R&D Systems, Bio-techne, Minneapolis, MN, USA, lower limit of detection: 6.23 pg/mL, assay range: 15.6–1000 pg/mL; MCP-1, R&D Systems, Bio-techne, Minneapolis, MN, USA, sensitivity: 10 pg/mL, assay range: 31.2–2000 pg/mL following the protocol for cell culture supernatants). Data acquisition was performed using a Multiskan™ SkyHigh Microplate Spectrophotometer (Thermo Fisher Scientific, Waltham, MA, USA) and analyzed using SkanIt™ Software RE for Microplate Readers, version 6.1.1.7 (Thermo Fisher Scientific, Waltham, MA, USA), with a 5-parameter model.

In the second part of the study, propensity score matching (1:3) was performed based on the main AKI risk factors: donor risk index (DRI), macrovesicular steatosis, and NaMELD recipient score. Since no graft displayed severe macrosteatosis, it was categorized as none/mild vs. moderate (i.e., macrovesicular steatosis < or >30%).

Both donation after brain death (DBD) and donation after circulatory death (DCD) were included. All procedures were conducted similarly, except for the cava anastomosis. Most of the grafts were reconstructed according to the piggyback technique, but in the case of HCC involving segment one and/or the caudate lobe encasing the cava, a classic technique was used without venovenous bypass. All recipients received the same immunosuppressive protocol.

The study was conducted in accordance with the ethical standards of our institutional Committee on Human Experimentation of our institution and reported according to the Strengthening the Reporting of Observational Studies in Epidemiology (STROBE) guidelines [38].

### 4.2. Definitions

Baseline serum creatinine was measured at the time of patient admission for transplantation. Post-transplant creatinine levels were assessed immediately upon transfer to the intensive care unit after orthotopic liver transplantation and subsequently on a daily basis. AKI was defined according to Kidney Disease Improving Global Outcomes (KDIGO) criteria: a serum creatinine value ≥ 1.5 times the pre-LT value within the first 7 days post-LT or an increase of 0.3 mg/dL within 48 h. Stage 1 was defined as an increase of 1.5 to 2 times baseline, stage 2 as 2–3 times, and stage 3 as >3 times baseline or the need for RRT [34].

The donor risk index was estimated according to Feng et al. [39].

Static-cold ischemia time (sCIT) in the MP group was defined as the time between cross-clamping during retrieval and initiation of MP. EAD was defined according to Olthoff’s criteria [40].

### 4.3. Modality of MP

The institutional protocol for MP included any DCD liver or any of the following donor risk conditions: donor age > 80 years, macrosteatosis ≥ 30%, estimated sCIT > 8 h, or expected long recipient hepatectomy.

The device was PerLiver (Aferetica S.r.l, Mirandola, Italy), applied as end-ischemic perfusion at the LT center. All grafts underwent dual hypothermic oxygenated ex situ perfusion for at least 2 h and until the completion of the recipient hepatectomy. The vessel perfusion was used at manually controlled pressure and flow. The arterial circuit was set up at 25–30 mmHg of pressure with pulsatile flow at 60 beats per minute and flow rates of 50–70 mL/min. Portal perfusion was continuous at 5 mmHg, with flow up to 500 mL/min. Temperature was maintained at 10–12 °C.

For DCD grafts, all cases were treated with normothermic regional perfusion followed by ex situ MP. Only grafts with macrosteatosis ≤ 30% and Ishak grade ≤ 1 at biopsy were considered suitable. National criteria for proceeding with liver retrieval under normothermic regional perfusion included ALT < 1000 UI/L (or a decreasing trend) and a decreasing lactate trend [41].

### 4.4. Statistical Analysis

Categorical variables are presented as counts and percentages, and differences between groups were assessed using Fisher’s exact or chi-square test, as appropriate. Continuous variables are expressed as mean ± standard deviation (SD) and compared using the independent-samples *t*-test. Normality was evaluated with the Kolmogorov–Smirnov test, and all variables showed a normal distribution.

Variables with a *p*-value < 0.5 in the univariate analysis were included in the multivariable logistic regression analyses of AKI. Hazard ratios (HRs) with 95% confidence intervals (CIs) were calculated.

Propensity score matching (PSM) (1:3, MP/SCS) was performed to minimize confounding. DRI, NaMELD score, and degree of steatosis were used as matching covariates. Logistic regression was used to estimate the propensity score. A no-replacement matched analysis was applied. Standardized mean differences (SMDs) < 0.1 were considered indicative of adequate balance.

Correlation between postoperative creatinine peak and MCP-1 or TNF-α levels was assessed using the Spearman correlation. Associations between AKI and cytokines were analyzed using logistic regression.

A two-tailed *p*-value < 0.05 was considered statistically significant. Analyses were performed using SPSS 24.0 (IBM Corp., Armonk, NY, USA), and propensity score matching was carried out using NCSS 2021 Statistical Software (NCSS, LLC, Kaysville, UT, USA).

## 5. Conclusions

In conclusion, despite the use of more marginal grafts, MP recipients did not experience worse renal outcomes after LT in terms of frequency and severity. Perfusate cytokine profiling may represent a promising early indicator of renal risk, deserving validation in larger prospective cohorts.

Future research should focus on ECD with severe macrosteatosis (>60%) or donors affected by significant infections, which currently remain major limiting factors for graft utilization. The safe use of these grafts with acceptable outcomes could further expand the donor pool, potentially beyond the gains already achieved through machine perfusion.

## Figures and Tables

**Table 1:** Data of donors, grafts, and recipients compared between the MP group and the SCS group before and after PSM analysis. (Abbreviations: CV: cardiovascular; DCD = donation after circulatory death; DRI = donor risk index; ICU = intensive care unit; LT = liver transplantation; MELD = model for end-stage liver disease; MP = machine perfusion; PSM = propensity score matching; SCS = static cold storage; WIT = warm ischemia time.)

		Before PSM			After PSM		
**Donor**	***Variables***	**MP** **(*n* = 30)**	**SCS** **(*n* = 147)**	***p-*** **Value**	**MP** **(*n* = 29)**	**SCS** **(*n* = 87)**	***p*** **-Value**
	**Age,** years	72 (±15)	62 (±15)	0.02	71 (±15)	64 (±13)	0.02
	**Gender,** male	13 (43)	78 (53)	0.42	13 (44.8)	49 (56.3)	0.29
	**Body mass index,** Kg/m^2^	28 (±9)	27 (±5)	0.68	28 (±9)	27 (±5)	0.79
	**Cause of death**			0.78			0.82
	Hypoxia	1 (3.3)	10 (6.8)		1 (3.4)	6 (6.9)	
	Trauma	3 (10)	19 (12.9)		3 (10.3)	7 (8)	
	CV accident	26 (87)	117 (79.6)		25 (86.2)	74 (85.1)	
	Others	0	1 (0.7)				
	**ICU stay,** days	4 (±4)	4 (±3)	0.63	4 (±3)	4 (±3)	0.63
	**DCD category 3**	3 (10)	0	0.04	2 (6.9)	0	0.06
	**DRI**	1.91 (±0.29)	1.82 (±0.34)	0.18	1.91 (±0.3)	1.89 (±0.82)	0.68
	**Diabetes**	9 (30)	23 (15.6)	0.72	8 (27.6)	9 (10.3)	0.03
	**CV disease**	20 (66.7)	82 (55.8)	0.31	20 (69)	50 (57.5)	0.38
**Grafts and Surgery**	**Macrosteatosis** **≥30%**	3 (10)	4 (2.7)	0.09	3 (10.3)	4 (4.6)	0.02
	**SCS,** min	435 (±135)	419 (±73)	0.51	433 (±137)	432 (±73)	0.97
	**WIT,** min	28 (±7)	32 (±32)	0.03	29 (+7)	31 (+10)	0.19
	**Piggyback**	26 (86.7)	127 (86.4)	1	25 (86.2)	76 (87.4)	1
**Recipient**	**Age,** years	58 (±9)	57 (±9)	0.77	58 (±10)	58 (±8)	0.95
	**Gender,** male	23 (77)	116 (79)	0.81	22 (75.9)	64 (73.6)	0.99
	**Body mass index,** Kg/m^2^	27 (±4)	26 (±6)	0.69	27 (±3)	26 (±6)	0.69
	**MELD**	16 (±7)	15 (±8)	0.38	16 (±7)	16 (±8)	0.82
	**Na MELD**	18 (±7)	16 (±9)	0.42	18 (±8)	18 (±8)	0.99
	**Pre-LT creatinine,** (mg/dL)	1.28 (±1.17)	0.97 (±0.69)	0.17	1.31 (±1.18)	1 (±0.78)	0.20
	**Liver disease**						
	Viral	11 (36)	55 (37.4)	0.89	10 (34.5)	32 (36.9)	1
	ALD	10 (33.3)	71 (48.3)	0.30	10 (34.5)	45 (51.7)	0.13
	Autoimmune	5 (16.7)	18 (12.2)	0.59	5 (17.2)	11 (12.6)	0.75
	MASH	12 (40)	39 (26.5)	0.24	11 (37.9)	24 (27.6)	0.50
	Others	3 (10)	15 (10.2)	0.91	3 (10.3)	8 (9.2)	1
	**Hepatocellular carcinoma**	11 (37)	39 (33.3)	0.86	10 (34.5)	25 (28.7)	0.64

**Table 2:** Postoperative outcomes in the MP group and the SCS group before and after PSM. (Abbreviations: AKI = acute kidney injury; ALT = alanine aminotransferase; AST = aspartate aminotransferase; ICU = intensive care unit; LT = liver transplantation; MP = machine perfusion; PSM = propensity score matching; SCS = static cold storage.)

		Before PSM			After PSM		
**Renal**	***Variables***	**MP** **(*n* = 30)**	**SCS** **(*n* = 147)**	***p*** **-Value**	**MP** **(*n* = 29)**	**SCS** **(*n* = 87)**	***p*** **-Value**
	**AKI**	19 (63.3)	67 (45.6)	0.16	17 (58.6)	41 (47.1)	0.39
	**Post-LT creatinine peak** (mg/dL)	1.96 (±1.02)	1.53 (±0.86)	0.02	2 (±1.02)	1.57 (±0.88)	0.03
	**AKI stage 1**	10 (33.3)	33 (22.4)	0.29	9 (31)	22 (25.3)	0.69
	**AKI stage 2**	5 (16.7)	20 (13.6)		5 (17.2)	13 (14.9)	
	**AKI stage 3**	4 (13.3)	14 (9.5)		4 (13.2)	8 (9.2)	
**Liver**	**EAD**	7 (23.3)	55 (37.4)	0.15	7 (24.1)	36 (41.4)	0.07
	**AST peak,** (mg/dL)	1252 (±1483)	785 (±1172)	0.16	1272 (±1506)	1686 (±1471)	0.20
	**ALT peak,** (mg/dL)	785 (±1177)	1172 (±1008)	0.07	801 (±1196)	1181 (±1002)	0.10
**Patient**	**ICU stay,** days	5 (±7)	3 (±7)	0.26	5 (±8)	4 (±10)	0.66
	**Hospital stay,** days	26 (±30)	18 (±17)	0.17	26 (±31)	19 (±21)	0.20
	**Mortality**	5 (16.7)	19 (12.9)	0.56	5 (17.2)	14 (16.1)	0.99

**Table 3:** Univariate and multivariate analysis of the donor, grafts, and recipient risk factors predicting AKI. (Abbreviations: AKI = acute kidney injury; CV = cerebrovascular disease; DCD = donation after circulatory death; DRI = donor risk index; ICU = intensive care unit; MELD = model for end-stage liver disease; MP = machine perfusion; SCS = static cold storage.)

		*Univariate*	*Multivariate*		
	**Variables**	***p*** **-Value**	***p*** **-Value**	**Odd Ratio**	**95% CI**
**Donor**	**Age**	0.94			
	**Gender**	0.88			
	**Body mass index**	0.54			
	**Cause of death**	0.95			
	**ICU stay**	0.87			
	**DCD**	0.60			
	**DRI**	0.99			
	**Diabetes**	0.03	0.009	3.29	(1.347–8.030)
	**CV disease**	0.88			
**Graft and surgery**	**Macrosteatosis** ≥30%	0.25			
	**MP**	0.16			
	**SCS,** min	0.34			
	**Warm ischemia time,** min	0.8			
	**Piggyback**	0.99			
**Recipient**	**Age**	0.02	0.006	1.06	(1.015–1.097)
	**Gender**	0.72			
	**Body mass index**	0.44			
	**MELD** **Na MELD**	0.72 0.87			
	**Cause of liver disease**	0.27			
	**Hepatocellular carcinoma**	0.58			

## Data Availability

The original contributions presented in this study are included in the article. Further inquiries can be directed to the corresponding author.

## Abbreviations

AKI	Acute Kidney Injury
DBD	Donation after Brain Death
DCD	Donation after Circulatory Death
DRI	Donor Risk Index
ECD	Extended Criteria Donor
ICU	Intensive Care Unit
IRI	Ischemia–Reperfusion Injury
MASH	Metabolic Dysfunction-Associated Steatohepatitis
MCP-1	Monocyte Chemoattractant Protein-1
MELD	Model of End-Stage Liver Disease
MP	Machine Perfusion
KDIGO	Kidney Disease: Improving Global Outcomes
LT	Liver Transplantation
PSM	Propensity score matching
RRT	Renal Replacement Therapy
SCS	Static Cold Storage
TNF-α	Tumor Necrosis Factor α

## References

[1] Jochmans I., Meurisse N., Neyrinck A., Verhaegen M., Monbaliu D., Pirenne J. (2017). Hepatic ischemia/reperfusion injury associates with acute kidney injury in liver transplantation: Prospective cohort study. Liver Transpl..

[2] Sharma P., Schaubel D.E., Guidinger M.K., Merion R.M. (2009). Effect of pretransplant serum creatinine on the survival benefit of liver transplantation. Liver Transpl..

[3] Klaus F., Keitel da Silva C., Meinerz G., Carvalho L.M., Goldani J.C., Cantisani G., Zanotelli M.L., Duro Garcia V., Keitel E. (2014). Acute kidney injury after liver transplantation: Incidence and mortality. Transplant. Proc..

[4] Rossi A.P., Vella J.P. (2016). Acute Kidney Disease After Liver and Heart Transplantation. Transplantation.

[5] Dong V., Nadim M.K., Karvellas C.J. (2021). Post-Liver Transplant Acute Kidney Injury. Liver Transpl..

[6] de Haan J.E., Hoorn E.J., de Geus H.R.H. (2017). Acute kidney injury after liver transplantation: Recent insights and future perspectives. Best. Pract. Res. Clin. Gastroenterol..

[7] Trinh E., Alam A., Tchervenkov J., Cantarovich M. (2017). Impact of acute kidney injury following liver transplantation on long-term outcomes. Clin. Transplant..

[8] Rubin A., Sanchez-Montes C., Aguilera V., Juan F.S., Ferrer I., Moya A., Montalva E., Pareja E., Lopez-Andujar R., Prieto M. (2013). Long-term outcome of ‘long-term liver transplant survivors’. Transpl. Int..

[9] Watt K.D., Pedersen R.A., Kremers W.K., Heimbach J.K., Charlton M.R. (2010). Evolution of causes and risk factors for mortality post-liver transplant: Results of the NIDDK long-term follow-up study. Am. J. Transplant..

[10] Thongprayoon C., Kaewput W., Thamcharoen N., Bathini T., Watthanasuntorn K., Lertjitbanjong P., Sharma K., Salim S.A., Ungprasert P., Wijarnpreecha K. (2019). Incidence and Impact of Acute Kidney Injury after Liver Transplantation: A Meta-Analysis. J. Clin. Med..

[11] Durand F., Francoz C., Asrani S.K., Khemichian S., Pham T.A., Sung R.S., Genyk Y.S., Nadim M.K. (2018). Acute Kidney Injury After Liver Transplantation. Transplantation.

[12] Leithead J.A., Rajoriya N., Gunson B.K., Muiesan P., Ferguson J.W. (2014). The evolving use of higher risk grafts is associated with an increased incidence of acute kidney injury after liver transplantation. J. Hepatol..

[13] Romano T.G., Schmidtbauer I., Silva F.M., Pompilio C.E., D’Albuquerque L.A., Macedo E. (2013). Role of MELD score and serum creatinine as prognostic tools for the development of acute kidney injury after liver transplantation. PLoS ONE.

[14] Lebron Gallardo M., Herrera Gutierrez M.E., Seller Perez G., Curiel Balsera E., Fernandez Ortega J.F., Quesada Garcia G. (2004). Risk factors for renal dysfunction in the postoperative course of liver transplant. Liver Transpl..

[15] Hilmi I.A., Damian D., Al-Khafaji A., Planinsic R., Boucek C., Sakai T., Chang C.C., Kellum J.A. (2015). Acute kidney injury following orthotopic liver transplantation: Incidence, risk factors, and effects on patient and graft outcomes. Br. J. Anaesth..

[16] Leithead J.A., Armstrong M.J., Corbett C., Andrew M., Kothari C., Gunson B.K., Muiesan P., Ferguson J.W. (2013). Hepatic ischemia reperfusion injury is associated with acute kidney injury following donation after brain death liver transplantation. Transpl. Int..

[17] Ito T., Naini B.V., Markovic D., Aziz A., Younan S., Lu M., Hirao H., Kadono K., Kojima H., DiNorcia J. (2021). Ischemia-reperfusion injury and its relationship with early allograft dysfunction in liver transplant patients. Am. J. Transplant..

[18] Umbro I., Tinti F., Scalera I., Evison F., Gunson B., Sharif A., Ferguson J., Muiesan P., Mitterhofer A.P. (2016). Acute kidney injury and post-reperfusion syndrome in liver transplantation. World J. Gastroenterol..

[19] Dar W.A., Sullivan E., Bynon J.S., Eltzschig H., Ju C. (2019). Ischaemia reperfusion injury in liver transplantation: Cellular and molecular mechanisms. Liver Int..

[20] Scalera I., Franzin R., Stasi A., Castellaneta A., Fischetti E., Morelli G., Raele M., Panetta E., Kurevija A., Pulga W. (2025). Haemoadsorption cartridge connected to the machine perfusion for donation after circulatory death porcine liver marginal grafts. World J. Transplant..

[21] Ghinolfi D., Lai Q., Dondossola D., De Carlis R., Zanierato M., Patrono D., Baroni S., Bassi D., Ferla F., Lauterio A. (2020). Machine Perfusions in Liver Transplantation: The Evidence-Based Position Paper of the Italian Society of Organ and Tissue Transplantation. Liver Transpl..

[22] Resch T., Cardini B., Oberhuber R., Weissenbacher A., Dumfarth J., Krapf C., Boesmueller C., Oefner D., Grimm M., Schneeberger S. (2020). Transplanting Marginal Organs in the Era of Modern Machine Perfusion and Advanced Organ Monitoring. Front. Immunol..

[23] De Carlis R., Di Sandro S., Lauterio A., Ferla F., Dell’Acqua A., Zanierato M., De Carlis L. (2017). Successful donation after cardiac death liver transplants with prolonged warm ischemia time using normothermic regional perfusion. Liver Transpl..

[24] Torri F., Balzano E., Melandro F., Maremmani P., Bertini P., Lo Pane P., Masini M., Rotondo M.I., Babboni S., Del Turco S. (2024). Sequential Normothermic Regional Perfusion and End-ischemic Ex Situ Machine Perfusion Allow the Safe Use of Very Old DCD Donors in Liver Transplantation. Transplantation.

[25] Patrono D., Cussa D., Sciannameo V., Montanari E., Panconesi R., Berchialla P., Lepore M., Gambella A., Rizza G., Catalano G. (2022). Outcome of liver transplantation with grafts from brain-dead donors treated with dual hypothermic oxygenated machine perfusion, with particular reference to elderly donors. Am. J. Transplant..

[26] Scalera I., De Carlis R., Patrono D., Gringeri E., Olivieri T., Pagano D., Lai Q., Rossi M., Gruttadauria S., Di Benedetto F. (2022). How useful is the machine perfusion in liver transplantation? An answer from a national survey. Front. Surg..

[27] Ghinolfi D., Rreka E., De Tata V., Franzini M., Pezzati D., Fierabracci V., Masini M., Cacciatoinsilla A., Bindi M.L., Marselli L. (2019). Pilot, Open, Randomized, Prospective Trial for Normothermic Machine Perfusion Evaluation in Liver Transplantation From Older Donors. Liver Transpl..

[28] van Rijn R., Endo C., Kucukerbil E.H., Blokzijl H., Blondeel J., Cortes Cerisuelo M., Coenraad M.J., Darwish Murad S., Doukas M., Eker H. (2025). Long-term Follow-up After Hypothermic Oxygenated Machine Perfusion in DCD Liver Transplantation: Results of a Randomized Controlled Multicenter Trial (DHOPE-DCD). Ann. Surg..

[29] Schlegel A., Mueller M., Muller X., Eden J., Panconesi R., von Felten S., Steigmiller K., Sousa Da Silva R.X., de Rougemont O., Mabrut J.Y. (2023). A multicenter randomized-controlled trial of hypothermic oxygenated perfusion (HOPE) for human liver grafts before transplantation. J. Hepatol..

[30] Panayotova G.G., Lunsford K.E., Quillin R.C., Rana A., Agopian V.G., Lee-Riddle G.S., Markovic D., Paterno F., Griesemer A.D., Amin A. (2024). Portable hypothermic oxygenated machine perfusion for organ preservation in liver transplantation: A randomized, open-label, clinical trial. Hepatology.

[31] Noren A., Molne J., Bennet W., Sorensen G., Herlenius G., Lindner P., Oltean M. (2023). End-ischemic hypothermic oxygenated machine perfusion does not improve renal outcome following liver transplantation from aged donors: A single-center retrospective report. Artif. Organs.

[32] Czigany Z., Pratschke J., Fronek J., Guba M., Schoning W., Raptis D.A., Andrassy J., Kramer M., Strnad P., Tolba R.H. (2021). Hypothermic Oxygenated Machine Perfusion Reduces Early Allograft Injury and Improves Post-transplant Outcomes in Extended Criteria Donation Liver Transplantation From Donation After Brain Death: Results From a Multicenter Randomized Controlled Trial (HOPE ECD-DBD). Ann. Surg..

[33] Avolio A.W., Spoletini G., Cillo U., Croome K., Oniscu G., Burra P., De Santibanes M., Egawa H., Gastaca M., Guo Z. (2025). Protocol for an international multicenter, prospective, observational, non-competitive, study to validate and optimise prediction models of 90-day and 1-year allograft failure after liver transplantation: The global IMPROVEMENT Study. Updates Surg..

[34] Khwaja A. (2012). KDIGO clinical practice guidelines for acute kidney injury. Nephron Clin. Pract..

[35] Pezzati D., Torri F., Franzini M., Balzano E., Catalano G., Tincani G., Bronzoni J., Martinelli C., Trizzino A., Petagna L. (2025). Association of perfusate cytokine concentrations during liver graft ex situ normothermic perfusion to donor type and postoperative outcomes. Liver Transpl..

[36] Friedman B.H., Wolf J.H., Wang L., Putt M.E., Shaked A., Christie J.D., Hancock W.W., Olthoff K.M. (2012). Serum cytokine profiles associated with early allograft dysfunction in patients undergoing liver transplantation. Liver Transpl..

[37] Pulitano C., Ho P., Verran D., Sandroussi C., Joseph D., Bowen D.G., McCaughan G.W., Crawford M., Shackel N. (2018). Molecular profiling of postreperfusion milieu determines acute kidney injury after liver transplantation: A prospective study. Liver Transpl..

[38] von Elm E., Altman D.G., Egger M., Pocock S.J., Gotzsche P.C., Vandenbroucke J.P., Iniciativa S. (2008). The Strengthening the Reporting of Observational Studies in Epidemiology (STROBE) statement: Guidelines for reporting observational studies. Rev. Esp. Salud Publica.

[39] Feng S., Goodrich N.P., Bragg-Gresham J.L., Dykstra D.M., Punch J.D., DebRoy M.A., Greenstein S.M., Merion R.M. (2006). Characteristics associated with liver graft failure: The concept of a donor risk index. Am. J. Transplant..

[40] Olthoff K.M., Kulik L., Samstein B., Kaminski M., Abecassis M., Emond J., Shaked A., Christie J.D. (2010). Validation of a current definition of early allograft dysfunction in liver transplant recipients and analysis of risk factors. Liver Transpl..

[41] De Carlis R., Lauterio A., Centonze L., Buscemi V., Schlegel A., Muiesan P., De Carlis L., Italian D.C.D.C.G. (2022). Current practice of normothermic regional perfusion and machine perfusion in donation after circulatory death liver transplants in Italy. Updates Surg..

